# Abdominal cocoon syndrome: a case report

**DOI:** 10.1093/jscr/rjaf696

**Published:** 2025-09-10

**Authors:** Fazeela Bibi, Muhammad Ibrahim, Uzair Khan, Shafiq Ur Rahman, Khalil El Abdi, Muhammad Aimal Khan, Sara Khizar, Usama Khan, Javed Iqbal, Umama Alam

**Affiliations:** Department of Medicine, Jinnah Medical and Dental College, Shaheed-e-Millat Road, Karachi East District, Karachi, Sindh 74800, Pakistan; Department of MBBS, Bannu Medical College, Bannu Township, Bannu District, Khyber Pakhtunkhwa 28100, Pakistan; Department of MBBS, Lady Reading Hospital, Soekarno Road, Peshawar Cantonment, Peshawar, Khyber Pakhtunkhwa 25000, Pakistan; Department of Medicine, Saidu Medical College, Saidu Sharif, Swat District, Khyber Pakhtunkhwa 19200, Pakistan; Faculty of Medicine and Pharmacy, Mohammed V University, Avenue Mohammed Belarbi El Alaoui, BP 6203, Rabat Institutions, Souissi, Rabat 10100, Morocco; Department of MBBS, Lady Reading Hospital, Soekarno Road, Peshawar Cantonment, Peshawar, Khyber Pakhtunkhwa 25000, Pakistan; Department of Medicine, Khyber Medical College, University Campus, Peshawar, Khyber Pakhtunkhwa 25120, Pakistan; Department of Medicine, Nowshera Medical College, Kaka Sahib Road, Nowshera District, Khyber Pakhtunkhwa 24100, Pakistan; Nursing Department, Hamad Medical Corporation Doha, PO Box 3050, Doha, Qatar; Department of Medicine, Khyber Medical College, University Campus, Peshawar, Khyber Pakhtunkhwa 25120, Pakistan

**Keywords:** abdominal cocoon syndrome, sclerosing encapsulated peritonitis, intestinal obstruction

## Abstract

Abdominal cocoon syndrome (ACS) is a rare cause of intestinal obstruction characterized by the encasement of the small intestine in a fibrocollagenous membrane, making preoperative diagnosis challenging. We present the case of a 15-year-old male with acute intestinal obstruction, where a contrast-enhanced computed tomography scan revealed a pathognomonic cocoon-like structure and concurrent gut malrotation. The diagnosis was confirmed intraoperatively, and management involved membrane excision, adhesiolysis, and a simple appendectomy for a secondarily involved appendix. This case is clinically significant for demonstrating tailored intraoperative decision-making; the appendicular involvement was correctly identified as a secondary consequence of the cocoon’s chronic inflammation, thus avoiding an unnecessary hemicolectomy. This report underscores ACS as a critical differential diagnosis for intestinal obstruction in young males, particularly in tropical regions, and highlights that early, pathology-guided surgical intervention is essential for favorable outcomes.

## Introduction

Abdominal cocoon syndrome (ACS) is a rare condition resulting in partial or complete intestinal obstruction. It involves the encasement of the small intestine in a dense, fibrocollagenous membrane, leading to impaired motility and obstruction. Histopathological examination reveals a fibrocollagenous membrane with inflammatory markers, critical in differentiating ACS from other conditions like peritoneal carcinomatosis and tuberculosis induced peritonitis [1]. Etiologically, ACS is categorized as primary (idiopathic) or secondary. Idiopathic ACS is prevalent in tropical and subtropical regions, primarily affecting young males, with a male-to-female ratio of 1:7. Secondary ACS arises from chronic peritoneal inflammation, linked to conditions such as tuberculosis, peritoneal dialysis, prior abdominal surgery, organ transplantation, or chronic medication use [2]. Preoperative diagnosis is challenging due to the nonspecific nature of symptoms, including abdominal pain, nausea, vomiting, and constipation. Imaging techniques, such as computed tomography (CT) and magnetic resonance imaging, often provide inconclusive results. Findings such as the “cauliflower sign” on small bowel contrast studies may aid diagnosis but lack consistency across cases [1]. In most instances, diagnosis is made intraoperatively through visualization of the fibrous membrane enveloping the intestines. Early surgical intervention remains the cornerstone of ACS management to prevent severe complications [2]. This report presents a case of idiopathic ACS with the rare concurrent finding of gut malrotation, and more significantly, provides a critical teaching point on surgical strategy: the correct interpretation of an appendicular mass as a secondary fibrotic manifestation of ACS, not a primary pathology, guided a proportionate and successful appendectomy over a more morbid hemicolectomy.

## Case description and investigations

A 15-year-old male presented to the surgical emergency department with acute abdominal pain, nausea, vomiting, and constipation persisting for 4 days. His medical history revealed no prior abdominal surgery, systemic illnesses, or prolonged medication use. Physical examination demonstrated abdominal tenderness and rigidity. Digital rectal examination was normal, and vital signs were stable.

**Figure 1:**
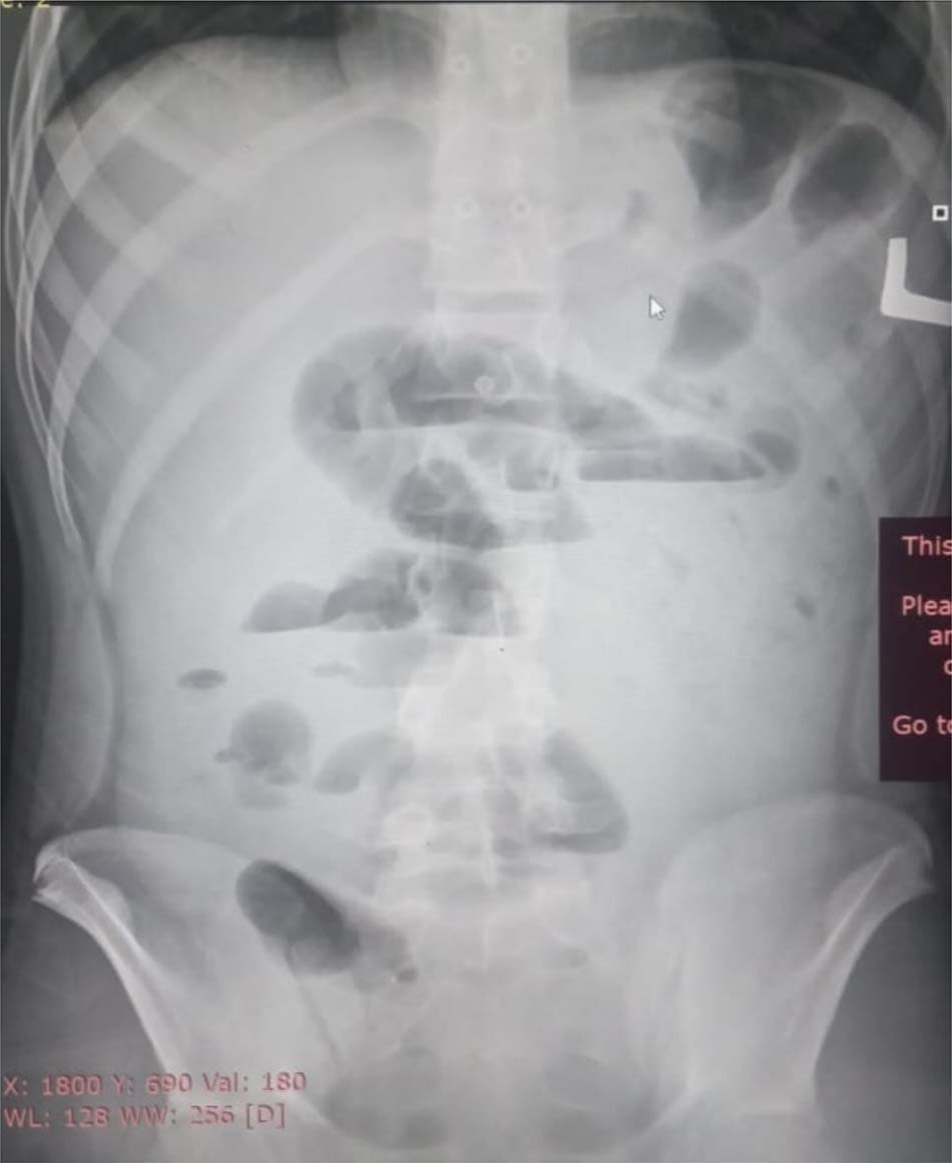
Initial radiographic evidence of bowel obstruction. This preoperative abdominal X-ray shows numerous air-fluid levels and significant distention of the bowel loops, confirming the clinical suspicion of intestinal obstruction.

**Figure 2:**
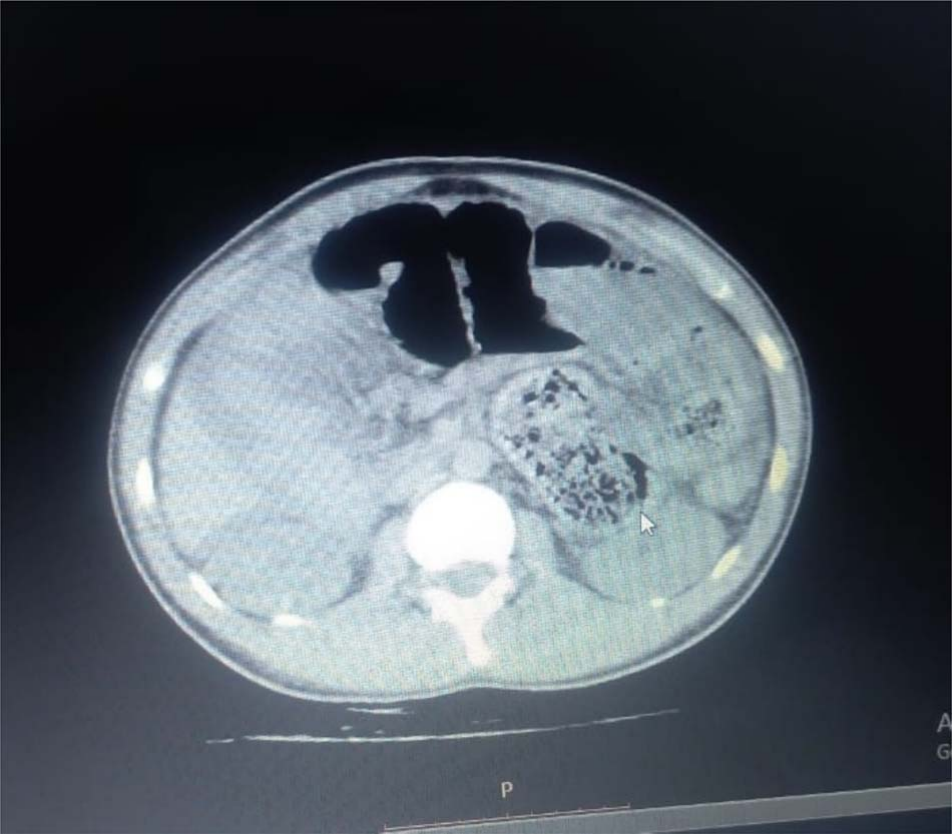
Axial CT demonstrating key pathological features. This contrast-enhanced axial view shows the colon displaced to the left side of the abdomen, confirming the presence of gut malrotation. The centrally clustered small bowel loops are seen encased in a dense sac, forming the characteristic cocoon structure that is pathognomonic for abdominal cocoon syndrome.

**Figure 3:**
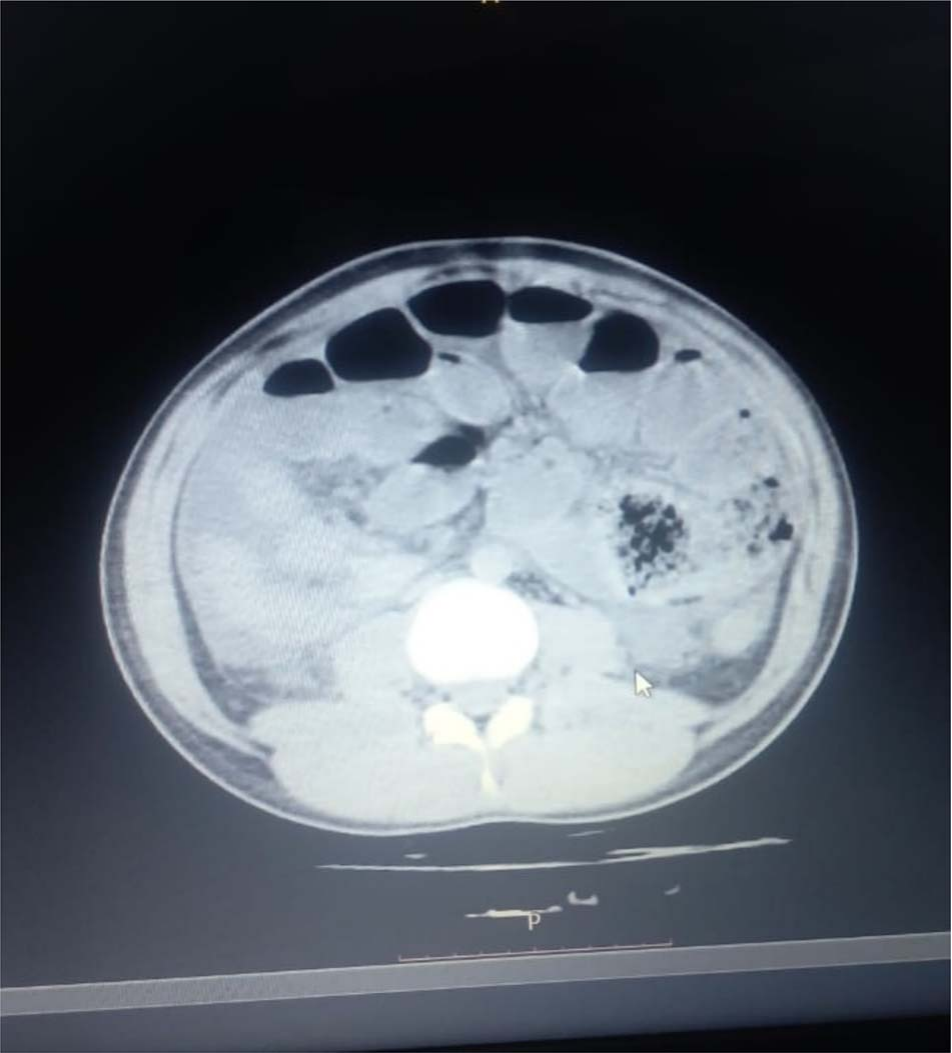
Abnormal positioning of encapsulated small bowel. This axial CT view, taken at a lower abdominal level, illustrates the profound anatomical derangement. The entire cluster of small bowel loops is seen encased and displaced into the right iliac fossa, a finding consistent with the combined effects of the cocoon membrane and the patient’s gut malrotation.

**Figure 4:**
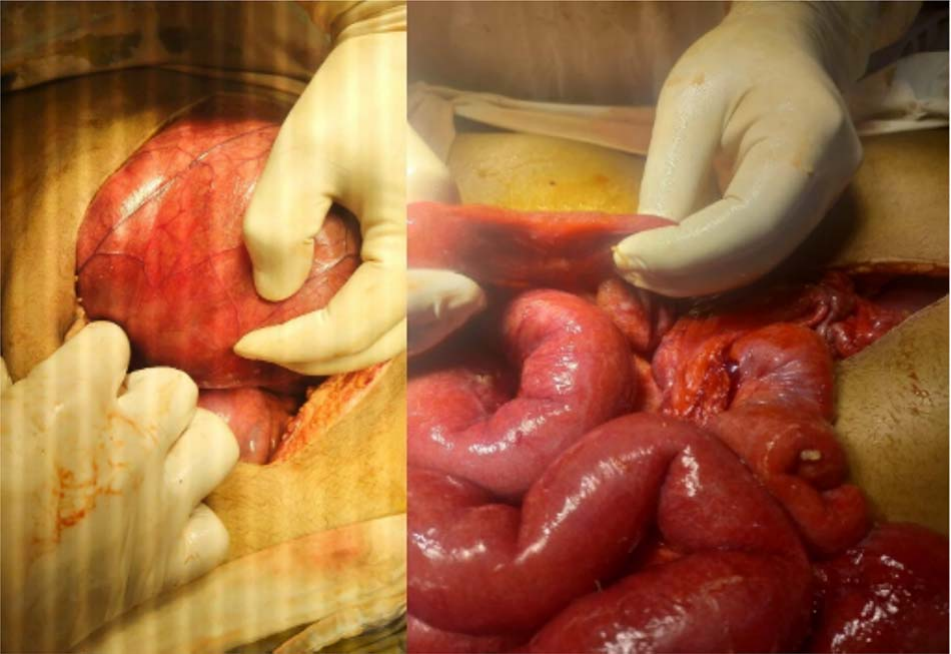
Intraoperative findings of idiopathic Sclerosing encapsulating peritonitis. The image displays the abdominal viscera entirely encased within a smooth, thick, cocoon-like sac, consistent with abdominal cocoon syndrome. The whitish, glistening appearance of the membrane is characteristic of its dense, fibrocollagenous composition, which was later confirmed by histopathology.

Initial radiographic evaluation with a plain abdominal X-ray confirmed the clinical suspicion of acute intestinal obstruction, demonstrating multiple air-fluid levels and diffuse small bowel dilatation (Fig. 1). To further delineate the underlying etiology, a contrast-enhanced computed tomography (CECT) scan was performed; providing critical diagnostic information it revealed, in the right iliac fossa, a striking encapsulation of the small intestine loops within a sac-like membrane, accompanied by ascites. This constellation of findings formed a pathognomonic cocoon-like structure (Fig. 2). Furthermore, the CECT identified a concurrent gut malrotation, evidenced by a left-sided displacement of the colon (Fig. 3). Laboratory findings showed an elevated total leukocyte count (TLC) of 21 000, indicative of inflammation, while other parameters were within normal limits. The patient underwent an exploratory laparotomy. Intraoperatively, the abdominal viscera were encased within a thick fibrous membrane (Fig. 4), containing ascitic fluid. The membrane was incised, adhesiolysis was performed, and the entrapped small bowel loops were released, and then a kink in the small intestine causing obstruction was identified and corrected. Additionally, the appendix was found to be secondarily involved in the encapsulating process. It was encased in the dense fibrocollagenous membrane, forming an inflammatory mass consistent with chronic serositis and localized fibrosis, rather than a classic acute appendicular phlegmon. Based on this intraoperative assessment that the mass was a manifestation of the cocoon’s chronic inflammation, a simple appendectomy was performed instead of a more extensive right hemicolectomy. This procedure was necessary to achieve complete release of the entrapped bowel, remove this localized inflammatory focus, and obtain a specimen for histopathology to definitively rule out a primary appendiceal pathology as a secondary cause of the peritonitis. Furthermore, ascitic fluid analysis showed no bacterial or mycobacterial growth, ruling out tuberculosis and the histopathological examination confirmed the fibrocollagenous nature of the membrane. The patient recovered well postoperatively and was discharged on the sixth day with advice for a follow-up after 2 weeks. At the follow-up visit, he reported no symptoms, and abdominal imaging confirmed the absence of obstruction. Histology further confirmed the benign fibrous nature of the membrane, with no signs of malignancy.

## Discussion

ACS, also referred to as encapsulating peritoneal sclerosis, was first described by Owtschinnikow in 1907 and later popularized by Foo *et al*. in 1978 [3, 4]. Its rarity, coupled with nonspecific clinical and radiological presentations, makes ACS a diagnostic challenge.

ACS typically presents as acute intestinal obstruction, characterized by symptoms such as abdominal pain, vomiting, and constipation. In the absence of prior abdominal surgeries or systemic illnesses, the case aligns with the idiopathic form of ACS [5]. Preoperative investigations often fail to yield conclusive results, as imaging modalities like CT scans and contrast studies may reveal features such as bowel loop clustering or ascites but are rarely definitive [6]. In this case, CT findings of small bowel encapsulation and gut malrotation strongly suggested ACS, later confirmed intraoperatively [1].

The etiology of ACS is multifactorial. Primary ACS occurs without an identifiable cause, predominantly in young males [2]. Secondary ACS is associated with chronic peritoneal irritation due to infections, dialysis, or prior surgeries. Inflammatory processes trigger fibrocollagenous membrane formation, leading to bowel encasement [7]. The absence of tuberculosis, malignancy, or surgical history in this patient supports the diagnosis of idiopathic ACS.

It is important to note that the definitive management for ACS is surgical, involving careful membrane excision and complete adhesiolysis to free the entrapped bowel [8]. As ACS is most often diagnosed intraoperatively, surgeons must be prepared to address unexpected findings based on the underlying pathology. The discovery of an appendicular mass in our patient is a prime example of such a scenario [9].

The decision to perform a simple appendectomy rather than a more extensive right hemicolectomy was based on a clear understanding of ACS pathophysiology. In fact, ACS is characterized by chronic, low-grade peritoneal inflammation that can extend to secondarily involve adjacent organs [1]. Therefore, the appendicular mass was not considered a primary appendiceal disease but rather a direct consequence of the cocoon’s fibroinflammatory process encasing the appendix. The term “mass” in this context was interpreted as adhesion-related fibrosis and chronic serositis [1], which is fundamentally different from the pathophysiology of an acute suppurative appendicitis or a primary appendiceal neoplasm, the main indications for considering a hemicolectomy. Given that the mass was understood to be a secondary manifestation of the primary disease, a simple appendectomy was the most proportionate and targeted intervention. This approach effectively removed the involved organ for histological confirmation and resolved the localized fibrotic focus without exposing the patient to the significantly higher risks of a bowel resection and anastomosis. This tailored strategy underscores a critical principle in managing ACS: surgical decisions should be guided by the unique pathology of the disease, which is one of chronic fibrosis rather than acute infection or malignancy [1, 2].

While certain conditions, such as advanced peritoneal carcinomatosis, may present with characteristic gross features intraoperatively, a definitive diagnosis relies on histopathology, remaining the gold standard for confirming ACS and formally distinguishing it from such mimics [10]. The presence of fibroblasts, lymphocytes, and plasma cells in the excised membrane, with an absence of malignant cells, highlights the chronic inflammatory process and confirms the benign nature of the cocoon [10]. Excluding peritoneal tuberculosis is particularly critical, as it is a well-known cause of secondary ACS and is endemic in many of the same tropical and subtropical regions where idiopathic ACS is prevalent [2]. Peritoneal TB can create a thick, fibrinous exudate that closely mimics the cocoon membrane of ACS [11]. Therefore, its exclusion requires a multi-pronged approach: negative ascitic fluid analysis for mycobacterial growth, as performed in our case, and meticulous histopathological review of the membrane to rule out the presence of caseating granulomas, which are pathognomonic for tuberculosis [11].

Furthermore, in this tropical setting, severe typhoid fever (enteric fever) should be considered in the differential diagnosis. Although less common, the severe intestinal inflammation associated with typhoid, particularly terminal ileitis and mesenteric lymphadenopathy, can lead to the formation of extensive adhesions [12]. These inflammatory changes could potentially evolve into a cocoon-like structure or present a clinical picture of intestinal obstruction that mimics ACS [2]. Given that idiopathic ACS predominantly affects young individuals in tropical regions, the demographic and geographic overlap makes typhoid a relevant, albeit rare, consideration.

Management of ACS hinges on early surgical intervention: adhesiolysis, membrane excision, and intestinal decompression are effective strategies [2], as demonstrated in this case. Delayed treatment can lead to complications such as adhesions, infections, or enterocutaneous fistulas [13]. While rare, postoperative recurrence is a potential concern. Recent studies suggest exploring adjuvant therapies like steroids or tamoxifen to manage inflammation and prevent recurrence, though evidence remains limited [14].

## Conclusion

ACS is a rare but significant cause of intestinal obstruction, often diagnosed intraoperatively due to nonspecific preoperative findings. This case underscores the importance of considering ACS as a differential diagnosis in young males presenting with intestinal obstruction, particularly in tropical regions. Early surgical intervention is pivotal for favorable outcomes. Histopathological analysis aids in confirming the diagnosis and ruling out secondary causes. Increased awareness and further research into the pathogenesis and management of ACS are essential for improving diagnostic accuracy and treatment efficacy.
